# Odorant Normative Data for Use in Olfactory Memory Experiments: Dimension Selection and Analysis of Individual Differences

**DOI:** 10.3389/fpsyg.2016.01267

**Published:** 2016-08-24

**Authors:** Andrew G. Moss, Christopher Miles, Jane V. Elsley, Andrew J. Johnson

**Affiliations:** Department of Psychology, Faculty of Science and Technology, Cognition and Cognitive Neuroscience Research Centre, Bournemouth UniversityPoole, UK

**Keywords:** olfaction, memory, normative, database, individual differences

## Abstract

The present study reports normative ratings for 200 food and non-food odors. One hundred participants rated odors across measures of verbalisability, perceived descriptive ability, context availability, pleasantness, irritability, intensity, familiarity, frequency, age of acquisition, and complexity. Analysis of the agreement between raters revealed that four dimensions, those of familiarity, intensity, pleasantness, and irritability, have the strongest utility as normative data. The ratings for the remaining dimensions exhibited reduced discriminability across the odor set and should therefore be used with caution. Indeed, these dimensions showed a larger difference between individuals in the ratings of the odors. Familiarity was shown to be related to pleasantness, and a non-linear relationship between pleasantness and intensity was observed which reflects greater intensity for odors that elicit a strong hedonic response. The suitability of these data for use in future olfactory study is considered, and effective implementation of the data for controlling stimuli is discussed.

## Introduction

Cross-modal comparison between olfactory memory and memory for other sensory modalities has produced mixed findings. Some studies have reported a pattern of memory consistent with other stimulus types (e.g., White and Treisman, 1997; Miles and Hodder, 2005; Johnson and Miles, 2007), whereas others have reported qualitatively different trends for olfactory stimuli (e.g., Reed, 2000; Johnson and Miles, 2009; Johnson et al., 2013). One possible interpretation of the latter finding is that olfactory memory differs qualitatively to that for other stimulus types and potentially resides within a separate olfactory-specific memory store (Andrade and Donaldson, 2007; Zelano et al., 2009).

An alternative explanation for the above disparity may relate to the criteria employed for odor selection. The characteristics of an odor can be an important determinant of memory performance, both quantitatively and qualitatively. Importantly, short of an *a priori* assessment of name-ability, there is limited control on the psychological characteristics of the odors. These odor characteristics may be of importance in determining cross-modal serial position function congruence, since the psychological distinctiveness of items (a somewhat ill-defined construct that can be influenced by perceptual familiarity) is argued to affect both the primacy and recency components of the serial position curve (Hay et al., 2007).

One method by which the perceptual experience of odors can be assessed is from ratings of the odors across various dimensions. Judgments of this nature are typically obtained via subjective ratings pre-test (Yeshurun et al., 2008), during encoding (Larsson et al., 2004b), or after the experiment through *post-hoc* data collection (Olsson et al., 2009). Indeed, there is some merit to collecting data this way, the most notable being mitigation of individual differences. For example, individual naming ability can allow tailored selection of odorants for use in subsequent memory and discrimination tasks (Rabin and Cain, 1984; Rabin, 1988). However, issues arise when tasks require novel presentation, and speeded encoding or recognition. In addition, these methods of odor stimuli categorization are often inconsistent, utilizing different scales and tasks, and resulting in these data rarely being used beyond the confines of the study in which they were collected. To this extent, the data are study-dependent. It is, therefore, desirable to have a reliable catalog of odors and normative data which will facilitate the use of odors in olfactory memory research. Accordingly, the present study attempts to provide data norms for a large set of commercially available odors, analogous to that produced for words (Coltheart, 1981), faces (Ebner et al., 2010), and objects (Yoon et al., 2004). Normative data in the verbal processing literature allows strict control of the orthographic, phonological, and psychological characteristics of words. An odor data analog will thereby enable researchers to both strictly control for, and manipulate, levels of psychological difference.

There is some limited precedence for the use of normative data for olfactory stimuli. The University of Pennsylvania Smell Identification Test (UPSIT; Doty et al., 1984b) is a clinical test of olfactory ability and uses 40 microencapsulated “scratch and sniff” odorants within a standardized test of olfactory function. The creation of this test includes normative data for familiarity, pleasantness, intensity, and irritability, and has been used extensively in olfactory research (Nguyen et al., 2012). However, the UPSIT is a test of olfactory dysfunction, where normal olfactory function would see naming of these highly familiar odors at, or near, ceiling. Employment of such a stimulus-set would provide limited variability in terms of familiarity and, potentially, encourage a memory strategy utilizing verbal labels. An alternative is to use odorants from the MONEX-40 (Freiherr et al., 2012), a test designed to detect differences in olfactory identification abilities in a normal population. However, the normative ratings from this study again focus only on familiarity, intensity, and pleasantness, and are limited to a relatively small set of 40 odorants.

Perhaps the closest attempt to a normative database for olfactory recognition tasks was reported by Sulmont et al. (2002). In this study, odors were rated in terms of familiarity, perceived complexity, and pleasantness by 24 French-speaking participants. Verbal identification was tested by selecting the name from a 68-item forced-choice list. These ratings were used to generate two familiar and two unfamiliar recognition sets of 18 odors. Interestingly, some perceptual overlap between dimensions was found with a significant positive correlation between pleasantness ratings and familiarity (*R*^2^ = 0.53), a negative correlation between complexity and familiarity (*R*^2^ = 0.65), and a positive correlation between notes (a different dimension of complexity) and familiarity (*R*^2^ = 0.30).

Further to the primary aim of providing a database of olfactory normative data, the present study aims to advance the use of normative databases in olfactory memory research in two ways. First, we consider whether subjective perceptual ratings of odors are suitable for use in a normative database. Individual differences are undoubtedly present in the perception of odors (Kaeppler and Mueller, 2013), and are perhaps more influential than for perception of verbal or object stimuli. If these individual differences exceed the differences obtained across the corpus of stimuli, it would suggest that tailoring odors to participants based on their ratings (Rabin, 1988) is a more effective method for stimulus control. Second, we consider the relationships between the dimensions within this database. As discussed in detail below, perceptual measures of olfactory stimuli are rarely independent, and the relationships between these dimensions should be considered when selecting stimuli for further tests.

### Selection of perceptual dimensions for study

The present study involved the collection of normative data across a large set of commercially available odors [food and non-food odors are used since (Gilbert and Greenberg, 1992) suggest that using food-related odors only may limit generalizability]. A large number of measures were selected based upon past work with odors and different modality normative databases. Scales and questions were presented without accompanying interpretation guidance. That is, participants were free to interpret each measure as they wished. Below we outline the justification for these measures.

#### Verbalisability

The first dimension concerns the extent to which odors can be named. Typically, variations in odorant nameability have seen important effects on recognition (cf. Zucco, 2003; Frank et al., 2011), and dissociated neural activations for odors that can or cannot be named are suggested to reflect a dedicated mechanism for processing un-named odors (Zelano et al., 2009). However, the name for an odor is an arbitrary construct which can include the source of the odor, a manufacturer name, or even a similar odor source it resembles. In addition, identification (and thus naming) of even familiar odors is often very poor (Lawless and Engen, 1977). As such, correct identification (the “veridical label”) is likely not important when considering the effect naming has on recognition, and its use for categorization may lead to an overestimation of the amount of “un-nameable” odors. Rather, any odor that has an identifying verbal label attached to it should be considered as utilizing verbal codes (and could conceivably be represented as a verbal, rather than olfactory, code), whilst only very broad categories, such as a basic hedonic label, should be classed as non-verbalisable (Jönsson et al., 2011). In the present task participants are required to attach any verbal label to each odor, which is then scored according to the specificity of this label. However, a caveat to using the quality of labeling as a verbalisability measure is that consistency of labeling is not considered. That is, the naming of odors may only be important in memory experiments if the names attributed to the stimuli are consistently reproduced (Frank et al., 2011; Cornell Kärnekull et al., 2015). Despite this, a clear effect of this assessment of labeling quality has been observed on working memory performance (Jönsson et al., 2011) and thus appears to provide a reasonable measure of the role of verbal labeling in memory.

#### Describe-ability

Participants are also required to rate each odor's describe-ability (on a 7-point scale). Participants typically exhibit over-confidence in their ability to correctly name odors despite poor naming performance (Jönsson and Olsson, 2003). Discrepancies between participants' perceived and actual ability might reflect the difficulties in accessing the name of an odor; a feeling of knowing termed the “tip of the nose” phenomenon (Lawless and Engen, 1977). However, the verbalisability score used in the present study is clearly a much more liberal criterion than odor naming ability. Since there is no “wrong” verbal label, ability to label the odor is perhaps likely to reflect the participants' awareness of an odor's description (which would presumably include labels). Thus, with this method we might expect a strong relationship between perceived descriptive ability and actual ability to generate verbal labels.

#### Context availability

The third dimension is context availability. This measure is closely related to concreteness (Altarriba et al., 1999), and refers to whether the odor can be easily associated to the context or circumstances with which the odor might appear. Whilst one might label this dimension imageability (i.e., the ability of the stimuli to evoke a mental image, Richardson, 1975), we avoid such a label to prevent conflation with perceptual imageability (i.e., imagining the perceptual experience of an odor, see Stevenson et al., 2007).

Our measure of context availability requires a 7-point rating of the ability to imagine the odor source. For example, the odor “lemon” may evoke an image of a lemon, or the odor “chlorine” may evoke an image of a swimming pool. For the latter, the odor (or in this case the context in which the odor is experienced) may be clearly imageable despite a poor ability to identify a source. It is possible, however, that this rating might again simply reflect the verbalisability of the odor, since an image is likely to result from the word that is associated with the odor.

#### Pleasantness

The hedonic rating of an odor features in many studies of odor perception and memory (Doty et al., 1984b; Sulmont et al., 2002; Dalton et al., 2008; Nguyen et al., 2012). These studies show that pleasant/unpleasant odors result in activations in dissociated brain regions (Rolls et al., 2003), and are a particularly pertinent factor in odor perception by non-experts (Yoshida, 1964). Hedonic determination is considered a key function in olfaction and is even suggested to represent the primary method of discrimination between odors (Schiffman, 1974). Importantly for odor recognition tasks, less pleasant odors have produced better overall recognition (Nguyen et al., 2012), indicating an important role of the dimension in how we represent odors in memory. This finding also makes it important to match pleasantness of odors when inspecting the effects of other dimensions on recognition. In the present study, participants are required to rate pleasantness on a 7-point scale.

#### Intensity

The fifth dimension, intensity, is also measured on a 7-point scale. Although perceived intensity of an odor is related to the concentration of the odorant (Cain, 1969; Berglund et al., 1971), it is also suggested to depend on experience-dependent factors (Ayabe-Kanamura et al., 1998; Distel et al., 1999). Specifically, the proposed degree of independence between intensity and pleasantness has varied from being entirely separate (Bensafi et al., 2002), to being related (Distel et al., 1999), or identical (Henion, 1971) constructs. Some studies have attempted to mitigate cross-condition differences in odor intensity by manipulating substance quantity (Stevenson et al., 2007) or via dilution (Sulmont et al., 2002). However, the odor intensity in the present experiment was allowed to vary between each odor, allowing investigation into its relationship with other factors across a broad range of intensities.

#### Irritability

The sixth dimension, and one potentially related to both intensity and pleasantness is the perceived irritability. An irritability measure is included in the normative data for odors in the UPSIT, and this measure would be expected to show a clear negative correlation with pleasantness as an additional reflection of a hedonic response. Irritability and pleasantness have shown differing effects on memory, where a recognition advantage for highly irritable odors is observed in older adults only (Larsson et al., 2009). Additionally, irritability has been used as an independently rated dimension when controlling high and low familiarity odor sets in memory tasks (Savic and Berglund, 2000). Whilst studies that do test irritability fail to clearly define this dimension, such a rating scale is likely interpreted as the physiological reaction to the odor. The findings by Larsson et al. (2009) indicate that our 7-point rating scale (very soothing/very irritating) will reveal a dimension that is independent of both pleasantness and intensity ratings.

#### Familiarity

The seventh measured dimension is familiarity. Odor familiarity is commonly a self-rated measure, though for verbal stimuli Brown and Watson (1987) suggest that subjective familiarity ratings are not a good substitute for objective frequency measures. This is because other factors such as frequency and age of acquisition ratings were found to contribute to judgments of familiarity (Brown and Watson, 1987). Despite this, such ratings of familiarity have been shown to be relatively stable when measured across different participants and time periods. For instance, ratings of familiarity from the UPSIT (Doty et al., 1984b) were utilized almost 30 years later in an odor memory study from Nguyen et al. (2012), and shown to correlate with new participant ratings (*r* = 0.46, *p* = 0.004). Similarly, Köster et al. (2002) compared familiarity scores provided for 12 odors with an earlier study (Degel et al., 2001) and found no significant differences in familiarity ratings.

#### Frequency

Familiarity is a complex construct which may be influenced by other dimensions. For example, word frequency is considered one of the most important variables in word processing (Brysbaert and New, 2009) and can be measured both objectively, via written or spoken appearances, and subjectively, via ratings of how often a particular word is experienced (Balota et al., 2004). The eighth dimension included is therefore of odorant frequency. Whilst an objective frequency measure for odors might, theoretically, be possible, subjective self-ratings are a more practical method of assessment. Such a rating scale is demonstrated with verbal stimuli to be a valid, and at times better, predictor of recognition performance than corpus frequency (Balota et al., 2004). Previous work by Sulmont et al. (2002) suggests that frequency and familiarity may be closely related (*R*^2^ > 0.85, *p* < 0.001). The present study will examine this through a 7-point rating scale.

#### Age of acquisition

A further construct that may influence familiarity (and our ninth measure in this study) is age of acquisition. Such a scale has not, to our knowledge, been studied previously for odors. It has, however, been shown to predict familiarity ratings and processing speed (Brown and Watson, 1987) for verbal stimuli. Age of acquisition for words is ideally mapped objectively by testing children on their naming ability, but has often been substituted for adult estimates of the age at which they first learnt the word. Morrison et al. (1997) suggest that these estimates can be a reliable and valid alternative measure if ratings (for example, because the sample are children) are unavailable. Our age of acquisition ratings will allow a first examination of odor age of acquisition and explore the relationship with familiarity ratings. Participants will simply state the age at which they first experienced the odor. Instances where participants believe an odor to be novel will be coded as the current age of that participant.

#### Perceived complexity

The tenth and final dimension assessed in the present study is perceived complexity. Perceived complexity will be measured subjectively, since analysis of the chemical complexity of odors have shown no relationship to their perceived complexity (Jellinek and Köster, 1983a). Subjective complexity ratings were shown to be reliable in a follow-up experiment, and as such are suggested to provide a meaningful measure in non-experts (Jellinek and Köster, 1983b). One might expect that ratings of an odor's complexity would relate to the perceivable odors that combine to make it; however, Sulmont et al. (2002) suggest there may be separable dimensions of complexity ratings and the perceived odor notes in an odor. They propose that perceived complexity is related to familiarity of the item, with complexity ratings reflecting the extent the stimuli can be interpreted as a meaningful unit. That is, a familiar odor will be rated as more simple. This is supported by a clear negative correlation of complexity with familiarity ratings. Alternatively, Jellinek and Köster (1983b) have previously shown no relationship between complexity and familiarity, but used a measure of “odor components” rather than a simple-complex rating scale. This question is presumably similar to the odor note question in Sulmont et al. (2002). It may be that an independent finding regarding “odor notes” comes from the ambiguity of this question for naïve participants. As such, complexity ratings in the present study focus on a scale of rated simplicity/complexity, on a 7-point Likert scale.

### Predictions

In utilizing a large number of odors in our normative study, we aim to obtain a wide range of scores across the dimensions. Across these dimensions, some interrelation is expected. Previous work (Sulmont et al., 2002) reported positive correlations between pleasantness and familiarity and a negative correlation between complexity and familiarity. Intuitively, one might expect correlations between measures of verbalization and prior exposure (e.g., familiarity, frequency, and age of acquisition), with the necessity for labels developing if one regularly encounters the stimuli. It is also prosaic to predict a negative correlation between pleasantness and irritability. This is the first study to try and assess age of acquisition (i.e., first exposure) for odors. However, if age of acquisition effects emulate that of verbal stimuli (see Morrison et al., 1997), one might expect age of acquisition to correlate negatively with familiarity, frequency, and context availability (i.e., the earlier that one is first exposed to the odor, the higher the ratings of familiarity, frequency, and imageability). Intensity is also expected to relate to pleasantness ratings, either as an increase in intensity as odors are rated unpleasant (Sezille et al., 2014), or perhaps an increase in intensity as pleasantness deviates from neutral (hedonic strength, Distel et al., 1999).

## Materials and methods

### Participants

One-hundred and three non-smoker students (18 male and 85 female, mean age = 19.4, age range = 18–34) were recruited via Bournemouth University's online experiment management system, and participated for course credits. Participants who self-reported olfactory impairments (for example, symptoms of cold) were excluded, as were participants aged older than 40 years. Age-based exclusion was due to the proposition that olfactory identification abilities peak between the third and fifth decade (Doty et al., 1984a; see also Wood and Harkins, 1987, for age-related differences in the recognition of odors). Three female participants withdrew from the study after the first session, leaving usable data from one hundred participants. This study was carried out with approval from the Bournemouth University ethics panel. All participants gave written informed consent in accordance with the Declaration of Helsinki.

### Design

A correlational design was used. The odors were grouped into 4 batches (A–D) of 50 odors (each containing 25 food and 25 non-food odors). Participants rated two of the four batches (that is, 100 odors) across two 60-min sessions separated by a minimum of 24-h. The presentation order of these batches was counterbalanced such that the testing orders A–B, B–A, C–D, and D–C were balanced across participants.

### Odorants

Two-hundred commercially available odorants (100 food-related and 100 non-food-related: see Appendix 1 in Supplementary Material for a complete list) were prepared by Dale Air Ltd. (www.daleair.com). These were stored within small test-tubes containing approximately 5ml of a liquid odorant soaked into a small piece of gauze. Due to contamination, odorant 17 (cabbage) was removed after 29 participant ratings. It remains included in our final database, but use of ratings for this odorant should be considered with caution.

### Procedure

Testing was undertaken in a well-ventilated and quiet laboratory. Participants were tested in groups varying in size from 2 to 8. In the test phase, odors were presented on test-tube trays containing a block of five odors, with each odor arbitrarily numbered from one to two-hundred. Within each testing session participants received 10 blocks of 5-odors, meaning participants smelled 50 odors in each of the two sessions. The composition of each 5-odor block was selected at random from the odor set within each batch. Participants were instructed to evaluate those odors in any order.

Evaluation required participants to open the test tube lid and smell the odor (birhinally) for approximately 3 s in order to answer each question. Between odors, participants took a break of approximately 20 s, and between odor blocks a break of 1 min was implemented where participants would take a drink of water. Responses were recorded within a booklet wherein each odor was assessed across the 10 dimensions. Ratings were measured on a 7-point Likert scale, labeled at each end, and at the neutral center point. Each dimension was identified from the literature discussed above, presented in the same order for each odor. “How *familiar* is this odor (not at all familiar/very familiar),” “how *intense* is this odor (very weak/very intense),” “how *pleasant* is this odor (very unpleasant/very pleasant),” “how *complex* is this odor (very simple/complex),” “how *irritating* is this odor (very soothing/very irritating),” “how *frequently* is this odor experienced (not at all frequently/very frequently),” “how easy is it to *describe* this odor (very difficult/very easy),” and “how easy is it to *imagine* where you'd experience this odor (very difficult/very easy).” In addition, two questions were open-ended. The first required a numerical age of acquisition response to “at what age did you first experience this odor?,” and the second a verbal written response to “can you attach any labels to this odor?” Participants were instructed to rate independently and in silence, and, if uncertain, participants were asked to guess.

## Results and discussion

### Scoring protocol

The first eight questions were coded on scales of 1–7 (familiarity, intensity, pleasantness, complexity, irritability, frequency, perceived describe-ability, and context availability).

In reporting age of acquisition, participants were encouraged to estimate the age at which an odor was first encountered, and provide a single age. When participants reported an age range as their answer, the median value of that range was recorded. A small number of participants provided a qualitative (rather than quantitative) age of acquisition response (for example, “childhood”). In this instance the age of acquisition score was not used.

The scoring of odor labels (verbalization) followed a modified version of the method described by Jönsson et al. (2011). These labels were coded on a 4-point scale (0–3). No response or a very basic affective judgment received a score of 0. Broad categorizations or generic labels (for example; cleaner, food, sweet) received a score of 1. More specific categorizations referring to specific groups (floral, perfume, sweets) received a score of 2, and any specific noun label received a score of 3. Scoring was performed independently by two researchers, with the median score taken as the final verbalisability value. Weighted Cohen's κ determined a good (Altman, 1991) level of agreement between raters, κ_w_ = 0.61 (95% CI, 0.59 to 0.62), *p* < 0.0005.

Responses were averaged across participants to give a normative score in every odorant for each dimension. The full normative ratings for the 200 odors can be found in Appendix 1 (Supplementary Material).

### Normative data reliability

In order for our normative data to be transferable to other samples in future studies, it is important to demonstrate that the variance in the ratings is attributable to the odors rather than individual differences in perception of the odors. Should the variance across participants match or exceed the variance between odors, it would suggest that tailoring odors according to individual participant ratings would be more suitable (Rabin, 1988).

In order to test this proposition we looked at each dimension individually and used an analytical method described by Uebersax (2015). As a measure of variability we examined the agreement of scores for each odor across our participants (individual differences). That is, for each dimension, we correlated an individual's rating of each odor with the average rating for that odor (a measure of “consistency across participants”). The higher the correlational coefficient, the greater the agreement between raters. Conversely, the lower the correlational coefficient, the greater the individual differences between raters. To assess the discriminability between odors, we correlated each individual's rating of an odor with their average rating across all odors for that dimension. A high correlation coefficient (a measure of “consistency across odors”) indicates little variation in the scores given for that dimension by each participant across odors. That is, participants respond similarly for that dimension across odors, indicating that the dimension is weak in discriminating between the odors. For the normative data in each dimension to be considered suitable we would expect the effect size for odor score agreement to significantly exceed that of rater score agreement. That is, ratings for an odor on each dimension should have a stronger relationship with the mean rating for that odor compared to the relationship to the mean rating across odors. To test this proposition we undertook a series of *t*-tests comparing the strength of effect size for the odor (consistency across participants) and the level of discriminability (consistency across odors) for each of the dimensions, which is shown in Table 1. As can be seen from the table, the effect sizes for these relationships differ across dimensions, so require some further consideration.

**Table 1:** **Mean *r* coefficients of rater agreement with each odor's mean score, and rating agreement with each rater's mean score**.

	**Dimension**									
	**Fam**.	**Pleas**.	**Int**.	**Comp**.	**Irr**.	**Freq**.	**CA**	**Desc**.	**AoA**	**Lab**
Consistency across participants	0.484	0.611	0.484	0.263	0.563	0.422	0.427	0.421	0.373	0.411
Consistency across odors	0.422	0.312	0.406	0.408	0.408	0.434	0.433	0.417	0.512	0.435
*t*-value	4.69^*^	21.69^*^	5.56^*^	−9.03^*^	10.37^*^	−0.16	−0.42	0.26	−9.32^*^	−1.48

* Comparisons significant to p < 0.001.

For ratings of familiarity, pleasantness, irritability, and intensity, the association of participants' responses to the mean response for an odor was significantly greater than the association of responses to the mean response for each participant. That is, responses for a particular odor were more closely associated to the normative score for that odor than they were to each participant's average response on that dimension. This suggests that those four dimensions are capable of discriminating between odors above any general response bias/strategy applied to that dimension. For complexity and age of acquisition, participants' ratings were more strongly related with the average rating for that dimension. This suggests a lack of sensitivity for complexity and age of acquisition. This finding may be due to several reasons. Participants may have shown little variability in how they respond for each odor, resulting in each response showing a strong relationship with the mean. For example, if they are unable to conceptualize “complexity” and “age of acquisition” they may adopt a default response for the question resulting in limited variability. Alternatively, a low association of ratings to each odor mean indicates a large effect of individual differences. Indeed, Table 1 shows that age of acquisition and complexity exhibited the lowest consistency across participants, indicating greater individual differences. These individual differences could occur through genuine variation in the ages at which an odor is first experienced or in the perceived complexity of odors, though may also arise from participant difficulties in interpreting and applying the particular question to the stimuli. Furthermore, ratings for frequency, context availability, and describe-ability, in addition to the labeling scores, showed no significant differences between the consistency across participants and consistency across odors. Consequently, these dimensions may exhibit reduced discriminatory power within a normative database.

### Relationships between dimensions

The linear correlation coefficient (*r*) was calculated for each dimension pairing, and displayed as a correlation matrix in Table 2. Almost all correlations were significant, with the exception of the intensity dimension with familiarity, frequency, describe-ability, context availability, and age-of-acquisition dimensions.

**Table 2:** **Correlation matrix (*r*) of averaged scores across participants for each odor**.

	**Q1**	**Q2**	**Q3**	**Q4**	**Q5**	**Q6**	**Q7**	**Q8**	**Q9**	**Q10**
Q1. Familiarity	–									
Q2. Intensity	0.05	–								
Q3. Pleasantness	0.73^*^	−0.53^*^	–							
Q4. Complexity	−0.40^*^	0.64^*^	−0.63^*^	–						
Q5. Irritability	−0.68^*^	0.61^*^	−0.98^*^	0.66^*^	–					
Q6. Frequency	0.92^*^	−0.08	0.77^*^	−0.50^*^	−0.73^*^	–				
Q7. Describe-ability	0.94^*^	0.09	0.67^*^	−0.42^*^	−0.62^*^	0.92^*^	–			
Q8. Context availability	0.95^*^	0.09	0.66^*^	−0.40^*^	−0.61^*^	0.93^*^	0.97^*^	–		
Q9. Age of acquisition	−0.91^*^	0.03	−0.72^*^	0.45^*^	0.69^*^	−0.88^*^	−0.89^*^	−0.90^*^	–	
Q10. Labeling score	0.88^*^	0.15^*^	0.54^*^	−0.28^*^	−0.50^*^	0.82^*^	0.88^*^	0.90^*^	−0.86^*^	–

* *Significant correlations at the 0.05 level*.

Some of the dimensional correlations warrant additional comment. As noted in the Introduction, this is the first study to attempt to assess the effect of age of acquisition in olfactory processing. Consistent, with the verbal domain (Morrison et al., 1997), we find that age of acquisition displays strong negative correlations with familiarity, frequency, and context availability. As expected, a strong negative correlation between age of acquisition and labeling was also reported, suggesting early exposure provides increased opportunities in which to develop a label for that odor. Indeed, intercorrelation was observed for several dimensions relating to knowledge and previous experience with the odorant. The strong relationship is present between these dimensions despite evidence that individual differences may exceed the variation observed across odors. Consequently, it's possible that these ratings may still have utility in a normative database, aiding researchers in odor selection before further tailoring of stimuli according to participant data.

**Figure 1:**
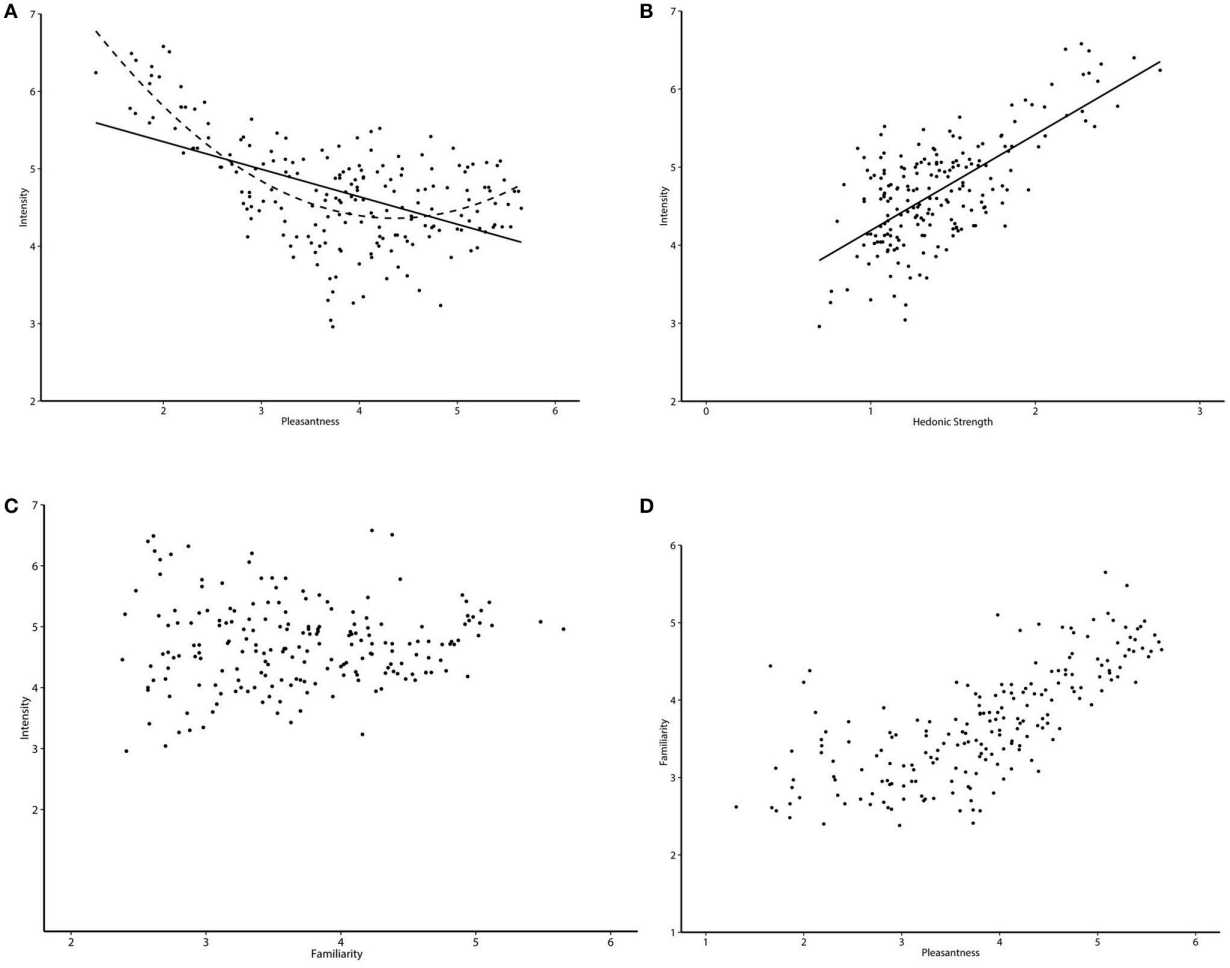
**Relationship of (A) pleasantness with intensity, (B) hedonic strength with intensity, (C) familiarity with intensity, and (D) pleasantness with familiarity**.

Of particular interest are the four dimensions identified as particularly suitable for use in a normative database; familiarity, pleasantness, irritability, and intensity. First, the strong negative correlation (*r* = −0.98) observed between irritability and pleasantness suggests collinearity, so further discussion focuses on only pleasantness scores. A predicted positive correlation between familiarity and pleasantness (Sulmont et al., 2002) was observed, and supports a classical mere-exposure effect (Zajonc, 1968). We predicted either a linear negative relationship between intensity and pleasantness (Sezille et al., 2014), or a non-linear relationship where intensity increases with both pleasantness and unpleasantness (Distel et al., 1999). In the present data, though a linear model was significant, *F*_(1, 198)_ = 75.23, *p* < 0.001, *R*^2^ = 0.28, a quadratic curve better fit the data, *F*_(2, 197)_ = 88.16, *p* < 0.001, *R*^2^ = 0.47 (Figure 1A). When pleasantness data were recoded as a measure of hedonic strength (with neutral responses scored as 0, increasing to 3 as they deviate above or below neutral), a linear model was accepted as the best fit, *F*_(1, 198)_ = 181.20, *p* < 0.001, *R*^2^ = 0.48 (Figure 1B). That is, intensity ratings are linearly related to the strength of a hedonic response. A strong relationship between hedonic strength and intensity supports ideas that the two may reflect similar dimensions of odor judgment (Henion, 1971).

However, a non-significant relationship between intensity and familiarity (Figure 1C) is an interesting result that is not consistent with the findings in Distel et al. (1999). They suggested that not only might an increased familiarity with a stimulus affect judgments of pleasantness (a relationship seen in our data, Figure 1D), but also that intense odors may be more easily recognized and thus more likely to be judged as familiar. Our observed pattern of relationships between familiarity, pleasantness, and intensity instead suggest that familiarity and intensity contribute independently to pleasantness scores.

## Discussion

The present study provides a large-scale normative dataset, containing ratings from 10 dimensions for 200 commercially available odors (see Appendix 1 in Supplementary Material). To date, this is the largest such study examining psychological dimensions for olfactory stimuli. These ratings are available in Appendix 1 (Supplementary Material) and provide the necessary information for researchers to control dimensions in subsequent studies (indeed, these data are part of a larger scale project examining factors which affect olfactory short-term memory, see Moss et al., unpublished). Additionally, our normative data is the first study to assess the effects of age of acquisition on olfactory processing. Whilst we show similar effects of age of acquisition to that shown with words (i.e., there was a strong negative correlation between age of acquisition and familiarity, Morrison et al., 1997), we showed that age of acquisition is strongly influenced by individual differences and that the dimension does not adequately differentiate between odors. As a result, the increased unsystematic variance in the age of acquisition norm values means they should be used with caution. Moreover, it is interesting to note that age of acquisition also exhibits strong negative correlations with frequency, describe-ability, context availability, and labeling score. Consequently, attempts to use the norms to isolate any effects of age of acquisition may be confounded by these inter-relations.

We suggest that our normative data provide two important benefits. The first benefit concerns experimental control. Since memory for odors has been shown to be affected by factors such as familiarity (e.g., Yeshurun et al., 2008) and pleasantness (Nguyen et al., 2012) we argue that it may be of utility to control for such issues analogously to that done with verbal memory. For example, if one were comparing memory for odors across two conditions (e.g., under conditions of quiet and concurrent articulation), matching the odors using our dimensions would eliminate a possible confound in that comparison. More specifically, studies examining serial position effects for odors report both differences across studies and potential qualitative differences with the functions reported for other stimulus types (Reed, 2000; Miles and Hodder, 2005; Johnson and Miles, 2007, 2009; Johnson et al., 2013). It is possible that these differences may be the effects of irregularities in the selection of stimuli; indeed, Hay et al. (2007) suggest that the psychological distinctiveness of stimuli can affect the shape of serial position curves. Our study provides a database from which researchers can systematically examine whether such serial position effect differences can be explained by characteristics of the odors. However, it should be noted that our data highlights some caveats in the selection of these dimensions since we find that only the norm ratings for familiarity, pleasantness, irritability, and intensity exhibit convincing discriminatory power. If researchers intend to investigate the effects of the remaining dimensions, it may be advisable to follow the approach undertaken by Rabin (1988), i.e., tailoring odors to individual ratings.

The second benefit of our normative dataset is that it provides a framework from which other researchers can examine the effects of psychological dimensions on olfactory memory. Researchers can use these data to explore whether dimensions that affect verbal memory similarly affect olfactory memory (as these odors are commercially available). One might expect that manipulating the familiarity of the stimulus set using our data would be of most interest in order to compare perceptual memory and the potential facilitative effects of verbal-perceptual dual-coding (Yeshurun et al., 2008).

That intensity was allowed to vary arguably reduces the usefulness of the normative data to the specific stimulus set used. It is possible that the relationship of intensity with pleasantness, and to some extent with irritability and complexity, may confound the scores obtained for these dimensions. We do not consider this a particular limitation, as the aim of the present study was to provide these data for a stimulus set that is readily-available and which does not require researchers to manually match odorant intensities to n-butanol. Selecting odorants for future research from the database can include matching odorants on intensity, whilst still allowing dimensions of interest to be manipulated. Furthermore, although several odors are artificially produced to reflect non-tangible objects (e.g., “sports locker room”), many of the odors are labeled from real-life objects. There is therefore opportunity for future research to expand the utility of these data by comparing other odor sources with the normative scores presented here.

Our normative data may be, to some extent, limited by our sample. The majority of participants were female (85%) and, in general, when sex differences are found in respect to olfaction females exhibit superiority (see Doty and Cameron, 2009, for review; although this trend can be complicated by menstrual cycle, e.g., Doty et al., 1981; Purdon et al., 2001). Of particular relevance to our normative data is the finding that females exhibit superior identification of odors (e.g., Larsson et al., 2004a). Indeed, Oberg et al. (2002) have shown that when naming ability is controlled, sex differences are removed (see also Larsson et al., 2003). That females are superior at naming odors may result in an inflation of the verbalisability score for our odors. Similarly, the use of university students in our sample may also have led to an overstatement of the name-ability of our odors. This is because educational level has been found to be a reliable predictor of odor identification (Moberg et al., 2014). Whilst our sample may have resulted, quantitatively, in a general inflation of ratings (particularly with respect to odor naming), there is no *a priori* reason to suggest that perception of these odors may have changed qualitatively with more males or a less educated sample. Consequently, we argue that the relative differences between the odors remains and the data retains its utility in differentiating odors. Notwithstanding, it is possible that our norms, particularly for food-based odors, may be limited cross-culturally. Gilbert and Greenberg (1992) question the universality of food-related odors since “what smells like food to persons of one culture may not smell edible to those of another” (p. 327). Different experiences with odors across cultures, both qualitatively and quantitatively, may fundamentally change conceptualization of those items. As a result, our norms may not translate to other cultures; although this is an empirically testable question that warrants further examination.

One might argue, however, that restricting the sample to a British-born student population functions to limit individual differences in the ratings of the odors, e.g., less culture-based variance in the preference for food-based odors (Kaeppler and Mueller, 2013). Notwithstanding this limitation in sample variance, we identify some dimensions that are less suitable for use in normative databases due to high levels of individual difference and/or a lack of sensitivity in discriminating between odors. For these dimensions a participant's average response across odors is more predictive of the rating than the average rating for that odor. This suggests dimension insensitivity. For these dimensions there was either a high level of variability between participants, or participants were conservative in the spread of scores they gave each odor. Interestingly, it is the dimensions that are most commonly considered in olfactory research that demonstrated most suitability for use in normative databases (those of familiarity, intensity, and pleasantness/irritability). However, the scales that did not meet the criterion of agreement should not be discounted. For example, our verbalisability scale was designed based on previous n-back research (Jönsson et al., 2011), and has shown working memory differences for odors selected based on this score. Indeed, other work from our laboratory has shown that odors which are high and low on verbalisability produce differences in proactive interference (Moss et al., unpublished) and olfactory n-back performance levels (Moss et al., unpublished). Further, correlations demonstrated between normative scores across dimensions, particularly those that have previously demonstrated relationships, support the validity of these scores. Therefore, rather than claiming that some of our dimensions lack utility, our data suggest that for some dimensions, individual differences/response biases may create more unsystematic variance in the normative values.

In summary, the normative data presented here may be utilized in future research to control odors for differences in olfactory perception. The dimensions should, however, be used with consideration of individual differences, particularly if testing a dissimilar population to that tested here. The ratings presented here do not offer a replacement for tailoring odors to participants (Rabin, 1988), but should be used where prior exposure of odors to participants is not desirable, or used to guide selection of odorants which can be later supplemented by *post-hoc* rating and categorization.

## Author contributions

AM: Contributed to the initial idea conception and study design, collected the data, performed the literature review, drafted the paper. AJ: Contributed to the study design, with substantial contribution to the interpretation of results, and critical revisions. JE: Contributed to the study design, contribution to interpretation of results, and critical revisions. CM: Contributed to the study design, contribution to interpretation of results, and critical revisions. All authors gave final approval of this work and agreement to be accountable for all aspects of the work.

### Conflict of interest statement

The authors declare that the research was conducted in the absence of any commercial or financial relationships that could be construed as a potential conflict of interest.
