# Supplementary material for: Odorant Normative Data for Use in Olfactory Memory Experiments: Dimension Selection and Analysis of Individual Differences
Source: Front Psychol. 2016 Aug 24;7:1267. doi: 10.3389/fpsyg.2016.01267 (PMC4995200; doi:10.3389/fpsyg.2016.01267)
Supplement: Supplementary file 1 [file DataSheet1.docx]

| Appendix 1. Normative data | | |  | |  | |  | |  | |  |  | |  | |  | |  | |  | |  | |  | |  |
| --- | --- | --- | --- | --- | --- | --- | --- | --- | --- | --- | --- | --- | --- | --- | --- | --- | --- | --- | --- | --- | --- | --- | --- | --- | --- | --- |
| Odor | Fam. | | Int. | | Pleas. | | Irr. | | Cont. Av. | | | Hed. Str. | | Comp. | | AoA | | Freq. | | Desc. | | Verb. | |  |  |  |
| Almonds | 5.34 | | 4.60 | | 5.12 | | 2.80 | | 4.28 | | | 1.36 | | 4.20 | | 9.03 | | 3.74 | | 4.16 | | 2.27 | |  |  |  |
| Aniseed Balls | 5.88 | | 5.40 | | 3.98 | | 3.54 | | 5.28 | | | 1.50 | | 4.08 | | 10.02 | | 4.22 | | 5.04 | | 2.61 | |  |  |  |
| Apples Green | 4.75 | | 4.21 | | 5.04 | | 2.83 | | 4.02 | | | 1.50 | | 3.62 | | 10.30 | | 3.83 | | 3.88 | | 2.04 | |  |  |  |
| Bacon | 3.15 | | 4.35 | | 2.90 | | 4.46 | | 2.65 | | | 1.27 | | 4.08 | | 14.93 | | 2.38 | | 2.21 | | 1.15 | |  |  |  |
| Banana | 5.40 | | 4.76 | | 5.32 | | 2.94 | | 4.54 | | | 1.56 | | 3.88 | | 9.89 | | 4.18 | | 4.48 | | 2.18 | |  |  |  |
| Barbecue | 3.62 | | 5.26 | | 2.30 | | 5.10 | | 3.14 | | | 2.02 | | 4.33 | | 13.44 | | 3.08 | | 2.98 | | 1.55 | |  |  |  |
| Basil | 4.06 | | 4.43 | | 4.12 | | 3.37 | | 3.35 | | | 1.22 | | 4.38 | | 12.66 | | 3.39 | | 2.96 | | 1.64 | |  |  |  |
| Beef | 3.80 | | 6.20 | | 1.88 | | 5.67 | | 3.47 | | | 2.33 | | 4.71 | | 13.88 | | 2.69 | | 3.41 | | 1.73 | |  |  |  |
| Biscuit | 4.17 | | 4.13 | | 3.48 | | 3.98 | | 3.50 | | | 1.31 | | 4.09 | | 12.17 | | 3.33 | | 3.25 | | 1.80 | |  |  |  |
| Blackberry | 5.04 | | 4.23 | | 5.23 | | 2.54 | | 4.48 | | | 1.52 | | 3.83 | | 9.66 | | 3.71 | | 4.46 | | 2.09 | |  |  |  |
| Blackcurrant | 5.67 | | 4.85 | | 5.48 | | 2.52 | | 5.13 | | | 1.73 | | 3.91 | | 7.12 | | 4.62 | | 4.67 | | 2.44 | |  |  |  |
| Blue Cheese | 3.51 | | 5.66 | | 1.89 | | 5.60 | | 2.87 | | | 2.19 | | 4.47 | | 14.29 | | 2.55 | | 2.94 | | 1.66 | |  |  |  |
| Brandy | 4.28 | | 4.60 | | 3.96 | | 3.92 | | 3.66 | | | 1.40 | | 3.92 | | 14.46 | | 3.64 | | 3.42 | | 1.76 | |  |  |  |
| Bubble Gum | 5.53 | | 5.04 | | 5.41 | | 2.49 | | 5.04 | | | 1.61 | | 4.19 | | 8.73 | | 4.27 | | 4.86 | | 2.34 | |  |  |  |
| Butter Cream | 5.02 | | 4.71 | | 4.35 | | 3.56 | | 4.08 | | | 1.23 | | 3.71 | | 10.91 | | 3.40 | | 3.79 | | 2.04 | |  |  |  |
| Buttered Popcorn | 2.72 | | 4.58 | | 3.02 | | 4.42 | | 2.80 | | | 1.47 | | 4.16 | | 15.07 | | 2.68 | | 2.70 | | 1.19 | |  |  |  |
| Cabbage | 3.00 | | 6.24 | | 1.31 | | 6.34 | | 2.69 | | | 2.76 | | 4.10 | | 15.32 | | 2.31 | | 2.48 | | 1.21 | |  |  |  |
| Candy Floss | 3.43 | | 4.04 | | 3.65 | | 4.00 | | 2.82 | | | 1.04 | | 3.88 | | 14.11 | | 2.90 | | 2.54 | | 1.38 | |  |  |  |
| Caramel Toffee | 4.16 | | 3.58 | | 4.28 | | 3.32 | | 3.46 | | | 1.24 | | 3.49 | | 11.84 | | 3.18 | | 3.30 | | 1.53 | |  |  |  |
| Cardamom | 4.08 | | 5.08 | | 3.32 | | 4.16 | | 2.92 | | | 1.52 | | 4.22 | | 13.51 | | 2.92 | | 2.84 | | 1.54 | |  |  |  |
| Carrot | 3.55 | | 4.69 | | 2.88 | | 4.45 | | 2.71 | | | 1.49 | | 3.90 | | 14.57 | | 2.67 | | 2.71 | | 1.29 | |  |  |  |
| Celery | 3.26 | | 4.70 | | 2.80 | | 4.86 | | 2.96 | | | 1.60 | | 4.24 | | 13.78 | | 2.78 | | 2.80 | | 1.59 | |  |  |  |
| Cereal | 4.17 | | 4.90 | | 3.29 | | 4.53 | | 3.96 | | | 1.37 | | 3.73 | | 11.40 | | 3.31 | | 3.45 | | 2.03 | |  |  |  |
| Cheddar Cheese | 3.14 | | 5.27 | | 2.35 | | 5.29 | | 2.79 | | | 1.86 | | 4.04 | | 15.90 | | 2.53 | | 2.63 | | 1.24 | |  |  |  |
| Cherry | 5.58 | | 4.76 | | 5.28 | | 2.78 | | 4.45 | | | 1.80 | | 3.73 | | 9.31 | | 3.88 | | 4.38 | | 2.28 | |  |  |  |
| Chewing Gum | 5.85 | | 5.02 | | 5.11 | | 3.04 | | 5.11 | | | 1.53 | | 3.75 | | 8.80 | | 4.81 | | 4.70 | | 2.64 | |  |  |  |
| Chicken | 3.40 | | 4.48 | | 2.85 | | 4.58 | | 2.81 | | | 1.69 | | 3.91 | | 13.74 | | 2.96 | | 2.67 | | 1.34 | |  |  |  |
| Chilli Pepper | 3.00 | | 4.14 | | 3.22 | | 4.29 | | 2.69 | | | 0.98 | | 4.25 | | 15.71 | | 2.54 | | 2.55 | | 1.31 | |  |  |  |
| Chocolate | 4.67 | | 4.41 | | 3.80 | | 3.37 | | 3.94 | | | 1.35 | | 3.98 | | 11.12 | | 3.82 | | 3.71 | | 2.06 | |  |  |  |
| Chocolate (Mint) | 4.90 | | 5.48 | | 4.12 | | 3.84 | | 4.22 | | | 1.32 | | 4.27 | | 11.11 | | 3.63 | | 4.06 | | 2.24 | |  |  |  |
| Chocolate (Orange) | 4.24 | | 5.04 | | 4.00 | | 3.74 | | 3.54 | | | 1.40 | | 4.35 | | 11.20 | | 3.44 | | 3.24 | | 1.88 | |  |  |  |
| Cinder Toffee | 3.02 | | 3.37 | | 3.59 | | 3.91 | | 2.26 | | | 1.04 | | 3.39 | | 14.80 | | 2.53 | | 2.47 | | 1.02 | |  |  |  |
| Cinnamon | 4.54 | | 5.00 | | 4.44 | | 3.52 | | 3.63 | | | 1.27 | | 4.26 | | 12.78 | | 3.50 | | 3.38 | | 1.79 | |  |  |  |
| Coco-mango | 5.27 | | 4.25 | | 5.55 | | 2.39 | | 4.61 | | | 1.64 | | 3.61 | | 10.42 | | 4.42 | | 4.20 | | 2.05 | |  |  |  |
| Coconut | 4.87 | | 3.23 | | 4.83 | | 2.85 | | 4.19 | | | 1.21 | | 3.59 | | 10.24 | | 3.94 | | 3.63 | | 2.15 | |  |  |  |
| Coffee | 4.45 | | 4.88 | | 3.76 | | 3.84 | | 4.24 | | | 1.47 | | 4.13 | | 10.97 | | 3.80 | | 3.82 | | 2.05 | |  |  |  |
| Cola | 5.21 | | 5.04 | | 5.02 | | 3.02 | | 4.29 | | | 1.35 | | 4.02 | | 10.05 | | 3.89 | | 3.81 | | 2.14 | |  |  |  |
| Cookie | 3.94 | | 3.94 | | 4.33 | | 3.45 | | 3.12 | | | 1.14 | | 4.19 | | 14.28 | | 2.94 | | 2.88 | | 1.35 | |  |  |  |
| Cookies & Cream | 4.30 | | 4.92 | | 3.80 | | 3.98 | | 3.80 | | | 1.28 | | 4.24 | | 12.70 | | 3.56 | | 3.64 | | 1.94 | |  |  |  |
| Coriander | 3.87 | | 4.76 | | 3.98 | | 3.87 | | 2.96 | | | 1.11 | | 3.98 | | 12.73 | | 2.98 | | 2.89 | | 1.51 | |  |  |  |
| Cranberry | 5.59 | | 4.71 | | 5.58 | | 2.59 | | 4.73 | | | 1.83 | | 3.88 | | 9.25 | | 4.55 | | 4.49 | | 2.29 | |  |  |  |
| Crusty Bread | 3.84 | | 3.76 | | 3.57 | | 3.76 | | 3.00 | | | 1.00 | | 3.69 | | 12.68 | | 3.58 | | 3.24 | | 1.38 | |  |  |  |
| Cucumber & Lime | 5.34 | | 4.28 | | 5.38 | | 2.72 | | 4.83 | | | 1.51 | | 3.91 | | 11.66 | | 4.55 | | 4.40 | | 2.34 | |  |  |  |
| Cumin | 4.27 | | 4.96 | | 3.30 | | 4.58 | | 3.46 | | | 1.44 | | 4.11 | | 13.13 | | 3.67 | | 3.00 | | 1.57 | |  |  |  |
| Curry | 4.81 | | 5.29 | | 3.79 | | 4.02 | | 3.77 | | | 1.29 | | 4.28 | | 11.70 | | 3.73 | | 3.40 | | 2.13 | |  |  |  |
| Fermented Fruit | 4.18 | | 5.24 | | 3.62 | | 4.14 | | 3.58 | | | 1.38 | | 4.49 | | 14.16 | | 3.20 | | 3.42 | | 1.77 | |  |  |  |
| Fish | 4.74 | | 5.78 | | 1.66 | | 5.66 | | 4.74 | | | 2.50 | | 3.69 | | 10.50 | | 3.68 | | 4.58 | | 2.57 | |  |  |  |
| Fruit Punch | 5.45 | | 4.18 | | 5.29 | | 2.49 | | 5.06 | | | 1.53 | | 3.77 | | 9.46 | | 4.63 | | 4.63 | | 2.45 | |  |  |  |
| Fruity Sweets | 5.02 | | 4.40 | | 5.04 | | 2.83 | | 4.38 | | | 1.38 | | 3.91 | | 10.65 | | 4.40 | | 4.02 | | 1.91 | |  |  |  |
| Garden Mint | 5.84 | | 5.08 | | 5.30 | | 2.32 | | 5.60 | | | 1.66 | | 3.77 | | 8.08 | | 5.24 | | 5.22 | | 2.62 | |  |  |  |
| Antiseptic | 3.48 | | 4.00 | | 3.30 | | 4.19 | | 3.35 | | | 1.13 | | 4.17 | | 13.04 | | 3.13 | | 3.08 | | 1.41 | |  |  |  |
| Baby powder | 5.48 | | 4.25 | | 5.46 | | 2.48 | | 4.67 | | | 1.63 | | 3.70 | | 9.83 | | 4.28 | | 4.25 | | 2.18 | |  |  |  |
| Beauty Soap | 4.98 | | 4.12 | | 4.71 | | 2.96 | | 4.71 | | | 1.33 | | 3.88 | | 9.05 | | 4.18 | | 4.31 | | 2.21 | |  |  |  |
| Black Pepper | 4.88 | | 5.04 | | 4.24 | | 3.66 | | 4.00 | | | 1.28 | | 4.18 | | 10.97 | | 3.84 | | 3.68 | | 1.77 | |  |  |  |
| Boiler Room | 3.04 | | 5.86 | | 2.42 | | 5.42 | | 2.60 | | | 1.94 | | 4.73 | | 14.96 | | 2.38 | | 2.51 | | 1.22 | |  |  |  |
| Brewery | 3.79 | | 3.94 | | 3.81 | | 3.58 | | 3.15 | | | 1.10 | | 3.62 | | 13.47 | | 3.23 | | 3.08 | | 1.69 | |  |  |  |
| Burning Peat | 3.81 | | 5.38 | | 2.79 | | 4.77 | | 3.54 | | | 1.54 | | 4.47 | | 12.99 | | 2.96 | | 3.11 | | 1.67 | |  |  |  |
| Burnt Wood | 3.90 | | 5.80 | | 2.18 | | 5.32 | | 3.78 | | | 1.98 | | 4.47 | | 12.44 | | 3.04 | | 3.24 | | 2.01 | |  |  |  |
| Cannon | 2.78 | | 6.40 | | 1.72 | | 5.90 | | 2.74 | | | 2.60 | | 5.10 | | 15.66 | | 2.22 | | 2.54 | | 1.34 | |  |  |  |
| Carbolic Soap | 3.61 | | 5.10 | | 3.12 | | 4.51 | | 2.92 | | | 1.33 | | 4.63 | | 14.33 | | 2.94 | | 2.94 | | 1.05 | |  |  |  |
| Casbah | 3.86 | | 4.70 | | 3.86 | | 3.88 | | 3.44 | | | 1.26 | | 4.45 | | 13.93 | | 3.16 | | 3.02 | | 1.63 | |  |  |  |
| Cedar | 3.68 | | 4.12 | | 3.36 | | 4.28 | | 3.34 | | | 1.12 | | 4.06 | | 13.21 | | 2.84 | | 3.08 | | 1.74 | |  |  |  |
| Church Incense | 4.08 | | 4.20 | | 3.64 | | 3.94 | | 3.38 | | | 1.12 | | 4.12 | | 13.10 | | 3.22 | | 3.06 | | 1.62 | |  |  |  |
| Cloisters | 3.41 | | 4.57 | | 3.14 | | 4.33 | | 3.00 | | | 1.22 | | 4.40 | | 13.93 | | 2.78 | | 2.61 | | 1.53 | |  |  |  |
| Clove Oil | 3.04 | | 5.02 | | 2.58 | | 4.60 | | 2.61 | | | 1.70 | | 4.43 | | 15.10 | | 2.47 | | 2.46 | | 1.24 | |  |  |  |
| Clover | 4.13 | | 4.02 | | 4.54 | | 3.14 | | 3.41 | | | 1.02 | | 4.00 | | 13.28 | | 3.26 | | 3.16 | | 1.63 | |  |  |  |
| Club | 3.12 | | 4.49 | | 3.20 | | 4.04 | | 2.78 | | | 1.29 | | 4.33 | | 14.38 | | 2.65 | | 2.49 | | 1.34 | |  |  |  |
| Coal Pit | 3.49 | | 6.49 | | 1.67 | | 5.96 | | 2.33 | | | 2.33 | | 5.08 | | 16.29 | | 2.14 | | 2.49 | | 1.26 | |  |  |  |
| Coal Soot | 4.27 | | 5.58 | | 2.46 | | 4.88 | | 3.69 | | | 1.88 | | 4.45 | | 12.19 | | 3.44 | | 3.48 | | 2.17 | |  |  |  |
| Cuban Cigar Smoke | 3.37 | | 4.52 | | 3.47 | | 4.04 | | 2.59 | | | 1.35 | | 4.60 | | 15.13 | | 2.61 | | 2.61 | | 1.05 | |  |  |  |
| Dentist | 3.66 | | 5.30 | | 2.88 | | 4.82 | | 3.16 | | | 1.68 | | 4.69 | | 13.83 | | 3.00 | | 2.90 | | 1.48 | |  |  |  |
| Earthy | 4.76 | | 4.88 | | 3.80 | | 3.76 | | 3.76 | | | 1.20 | | 4.55 | | 11.68 | | 3.52 | | 3.22 | | 1.78 | |  |  |  |
| Eau de Cologne | 4.92 | | 4.92 | | 4.43 | | 3.45 | | 3.78 | | | 1.08 | | 4.33 | | 11.59 | | 3.94 | | 3.65 | | 1.91 | |  |  |  |
| Eucalyptus | 5.88 | | 5.42 | | 4.73 | | 3.13 | | 4.96 | | | 1.06 | | 4.49 | | 9.90 | | 4.25 | | 4.63 | | 2.45 | |  |  |  |
| Fabric Softener | 4.90 | | 4.27 | | 4.67 | | 3.02 | | 4.29 | | | 1.29 | | 3.75 | | 10.60 | | 4.18 | | 4.20 | | 2.11 | |  |  |  |
| Farmyard | 3.86 | | 5.40 | | 2.46 | | 5.04 | | 3.76 | | | 2.06 | | 4.27 | | 12.24 | | 3.14 | | 3.10 | | 1.57 | |  |  |  |
| Firework | 3.71 | | 5.77 | | 2.32 | | 5.40 | | 2.81 | | | 2.06 | | 4.85 | | 14.73 | | 2.56 | | 2.79 | | 1.59 | |  |  |  |
| Football Pitch | 4.52 | | 5.00 | | 3.90 | | 3.75 | | 3.81 | | | 1.19 | | 4.19 | | 12.17 | | 3.67 | | 3.38 | | 1.85 | |  |  |  |
| Forest | 4.14 | | 4.04 | | 4.18 | | 3.46 | | 3.62 | | | 1.06 | | 3.84 | | 12.55 | | 3.40 | | 3.34 | | 1.71 | |  |  |  |
| Fox | 4.30 | | 4.62 | | 3.76 | | 3.84 | | 3.50 | | | 1.08 | | 4.04 | | 12.32 | | 3.18 | | 2.94 | | 1.75 | |  |  |  |
| Frosty | 4.00 | | 3.62 | | 4.49 | | 3.55 | | 3.70 | | | 1.30 | | 3.98 | | 13.93 | | 3.60 | | 3.49 | | 1.77 | |  |  |  |
| Gambia | 4.58 | | 4.13 | | 4.21 | | 3.13 | | 3.60 | | | 1.08 | | 3.79 | | 11.34 | | 3.38 | | 3.25 | | 1.83 | |  |  |  |
| Garden Shed | 4.04 | | 4.56 | | 3.81 | | 3.98 | | 3.46 | | | 1.10 | | 4.23 | | 12.81 | | 3.17 | | 3.06 | | 1.59 | |  |  |  |
| Ginseng | 3.94 | | 5.06 | | 3.02 | | 4.41 | | 3.10 | | | 1.43 | | 4.27 | | 14.59 | | 3.00 | | 2.55 | | 1.58 | |  |  |  |
| Grass/Hay | 4.98 | | 4.89 | | 4.04 | | 3.72 | | 4.09 | | | 1.02 | | 4.17 | | 11.18 | | 3.83 | | 3.55 | | 2.01 | |  |  |  |
| Havana Cigar | 4.76 | | 4.46 | | 4.28 | | 3.80 | | 3.86 | | | 1.16 | | 4.19 | | 11.57 | | 3.61 | | 3.48 | | 1.80 | |  |  |  |
| Hawthorn | 4.37 | | 4.14 | | 4.39 | | 3.45 | | 3.35 | | | 1.00 | | 4.02 | | 11.12 | | 3.61 | | 3.37 | | 1.94 | |  |  |  |
| Heather/Bracken | 5.12 | | 4.72 | | 4.60 | | 3.12 | | 4.46 | | | 1.16 | | 3.90 | | 10.96 | | 3.98 | | 3.94 | | 2.03 | |  |  |  |
| Honeysuckle | 3.98 | | 3.77 | | 4.00 | | 3.45 | | 3.42 | | | 1.17 | | 3.87 | | 12.93 | | 3.54 | | 3.25 | | 1.68 | |  |  |  |
| Hospital Modern Day | 5.06 | | 4.86 | | 4.32 | | 3.44 | | 4.10 | | | 1.00 | | 4.27 | | 11.36 | | 4.00 | | 3.66 | | 2.02 | |  |  |  |
| Hot Stuff Male | 4.62 | | 4.26 | | 4.76 | | 3.00 | | 4.06 | | | 1.36 | | 3.94 | | 12.81 | | 3.90 | | 3.86 | | 1.75 | |  |  |  |
| Hunter | 3.73 | | 3.85 | | 4.13 | | 3.52 | | 3.42 | | | 0.92 | | 3.81 | | 13.79 | | 3.48 | | 3.27 | | 1.45 | |  |  |  |
| Lavender | 4.84 | | 4.73 | | 4.67 | | 3.25 | | 4.51 | | | 1.16 | | 4.08 | | 10.84 | | 3.78 | | 4.20 | | 2.28 | |  |  |  |
| Leather | 3.88 | | 5.12 | | 3.24 | | 4.31 | | 3.35 | | | 0.96 | | 4.54 | | 12.96 | | 3.02 | | 3.02 | | 1.83 | |  |  |  |
| Leather Cream | 4.37 | | 3.92 | | 3.55 | | 3.59 | | 3.96 | | | 1.10 | | 4.10 | | 11.56 | | 3.27 | | 3.41 | | 1.50 | |  |  |  |
| Leather/Hide | 3.60 | | 4.28 | | 3.55 | | 4.00 | | 3.13 | | | 1.09 | | 4.35 | | 14.14 | | 2.85 | | 2.89 | | 1.46 | |  |  |  |
| Leaves | 4.80 | | 4.98 | | 4.02 | | 3.71 | | 4.27 | | | 1.20 | | 3.94 | | 11.33 | | 3.90 | | 3.84 | | 2.04 | |  |  |  |
| Lemon Cream | 5.64 | | 5.10 | | 5.44 | | 2.58 | | 4.96 | | | 1.60 | | 3.71 | | 10.77 | | 4.58 | | 4.62 | | 2.51 | |  |  |  |
| Mahogany | 3.27 | | 4.55 | | 2.82 | | 4.67 | | 2.50 | | | 1.47 | | 4.02 | | 14.85 | | 2.72 | | 2.23 | | 1.18 | |  |  |  |
| Man-o'-War | 3.14 | | 6.10 | | 1.86 | | 5.73 | | 2.44 | | | 2.38 | | 4.82 | | 15.10 | | 2.18 | | 2.86 | | 1.20 | |  |  |  |
| Garlic | 5.10 | | 6.51 | | 2.06 | | 5.51 | | 4.47 | | | 2.18 | | 4.21 | | 10.91 | | 3.94 | | 4.00 | | 2.30 | |  |  |  |
| Ginger | 3.39 | | 5.22 | | 3.10 | | 4.39 | | 3.00 | | | 1.39 | | 4.78 | | 13.93 | | 2.57 | | 2.84 | | 1.66 | |  |  |  |
| Gingerbread | 3.69 | | 4.04 | | 3.51 | | 3.79 | | 2.79 | | | 1.09 | | 4.19 | | 13.74 | | 2.73 | | 2.60 | | 1.59 | |  |  |  |
| Grapefruit | 4.22 | | 4.96 | | 4.18 | | 3.63 | | 3.88 | | | 1.08 | | 4.24 | | 12.77 | | 3.61 | | 3.33 | | 2.06 | |  |  |  |
| Hazelnut | 4.40 | | 5.40 | | 3.25 | | 4.31 | | 3.35 | | | 1.79 | | 4.27 | | 11.51 | | 3.23 | | 3.19 | | 1.95 | |  |  |  |
| Herring | 4.02 | | 4.64 | | 2.88 | | 4.46 | | 3.76 | | | 1.68 | | 3.90 | | 11.50 | | 3.22 | | 3.32 | | 1.99 | |  |  |  |
| Honey | 4.50 | | 4.10 | | 4.24 | | 3.34 | | 3.70 | | | 1.16 | | 3.80 | | 10.22 | | 3.38 | | 3.36 | | 2.07 | |  |  |  |
| Ice Cream | 3.13 | | 3.04 | | 3.71 | | 3.71 | | 2.56 | | | 1.21 | | 3.58 | | 14.29 | | 2.63 | | 2.50 | | 1.55 | |  |  |  |
| Iced Lemon | 5.71 | | 4.71 | | 5.63 | | 2.40 | | 4.69 | | | 1.96 | | 4.21 | | 8.05 | | 4.15 | | 4.47 | | 2.34 | |  |  |  |
| Irish Cream | 4.63 | | 4.31 | | 4.02 | | 3.58 | | 3.92 | | | 1.10 | | 4.25 | | 14.62 | | 3.50 | | 3.56 | | 2.10 | |  |  |  |
| Jelly Beans | 3.75 | | 3.73 | | 4.40 | | 3.11 | | 2.85 | | | 1.23 | | 3.81 | | 12.07 | | 2.96 | | 2.77 | | 1.74 | |  |  |  |
| Lemon, Eucalyptus & Mint | 5.67 | | 4.78 | | 4.76 | | 3.00 | | 4.67 | | | 1.12 | | 3.88 | | 10.07 | | 4.67 | | 4.45 | | 2.59 | |  |  |  |
| Lime | 5.70 | | 5.06 | | 5.16 | | 2.98 | | 5.18 | | | 1.40 | | 3.80 | | 8.28 | | 4.38 | | 4.88 | | 2.73 | |  |  |  |
| Liquorice | 5.14 | | 4.72 | | 3.56 | | 4.12 | | 4.40 | | | 1.28 | | 4.04 | | 12.59 | | 3.52 | | 3.88 | | 2.22 | |  |  |  |
| Lychee | 4.38 | | 4.50 | | 4.44 | | 3.33 | | 3.75 | | | 1.56 | | 3.90 | | 11.77 | | 3.25 | | 3.19 | | 1.75 | |  |  |  |
| Malted Barley | 3.20 | | 3.58 | | 3.70 | | 3.82 | | 2.75 | | | 1.34 | | 4.02 | | 13.57 | | 2.74 | | 2.74 | | 1.65 | |  |  |  |
| Mango & Sweet Orange | 5.02 | | 4.33 | | 5.12 | | 2.80 | | 4.29 | | | 1.49 | | 3.92 | | 9.17 | | 3.96 | | 4.13 | | 2.28 | |  |  |  |
| Mango Delight | 4.40 | | 4.42 | | 3.90 | | 3.53 | | 3.70 | | | 1.70 | | 3.92 | | 12.60 | | 3.20 | | 3.62 | | 1.83 | |  |  |  |
| Marzipan | 6.12 | | 5.27 | | 4.96 | | 3.00 | | 5.14 | | | 1.65 | | 4.22 | | 7.88 | | 4.00 | | 4.90 | | 2.73 | |  |  |  |
| Melon | 5.37 | | 4.55 | | 5.39 | | 2.92 | | 3.96 | | | 1.67 | | 4.35 | | 11.50 | | 3.71 | | 3.90 | | 2.20 | |  |  |  |
| Mixed Spice | 4.42 | | 4.88 | | 4.04 | | 3.92 | | 3.72 | | | 1.40 | | 4.45 | | 12.17 | | 3.50 | | 3.42 | | 1.97 | |  |  |  |
| Mulled Wine | 5.08 | | 3.94 | | 5.14 | | 2.68 | | 3.94 | | | 1.46 | | 3.84 | | 11.12 | | 4.26 | | 3.74 | | 2.01 | |  |  |  |
| Onion | 4.66 | | 6.58 | | 2.00 | | 6.02 | | 4.22 | | | 2.28 | | 4.46 | | 11.34 | | 3.82 | | 4.20 | | 2.28 | |  |  |  |
| Orange & Cinnamon | 5.28 | | 4.72 | | 5.10 | | 2.80 | | 4.34 | | | 1.54 | | 3.86 | | 10.49 | | 4.20 | | 4.20 | | 2.33 | |  |  |  |
| Orange (Seville) | 5.47 | | 4.45 | | 5.20 | | 2.61 | | 4.69 | | | 1.61 | | 3.86 | | 9.47 | | 4.33 | | 4.47 | | 2.30 | |  |  |  |
| Parma Violets | 4.73 | | 3.85 | | 4.94 | | 2.71 | | 3.92 | | | 1.40 | | 3.92 | | 11.89 | | 3.46 | | 3.65 | | 1.97 | |  |  |  |
| Passion Fruit | 4.69 | | 4.20 | | 4.82 | | 3.00 | | 4.14 | | | 1.55 | | 4.04 | | 10.93 | | 3.71 | | 3.55 | | 1.99 | |  |  |  |
| Peach Flesh | 5.33 | | 3.98 | | 5.20 | | 2.55 | | 4.14 | | | 1.41 | | 3.80 | | 10.43 | | 3.72 | | 4.02 | | 2.34 | |  |  |  |
| Peach Schnapps | 5.73 | | 4.49 | | 5.65 | | 2.59 | | 4.57 | | | 1.69 | | 3.65 | | 8.77 | | 4.06 | | 4.22 | | 2.40 | |  |  |  |
| Peanut | 3.85 | | 4.96 | | 2.74 | | 4.74 | | 3.32 | | | 1.85 | | 4.38 | | 12.52 | | 2.85 | | 3.09 | | 2.01 | |  |  |  |
| Pear | 5.82 | | 5.16 | | 4.40 | | 3.56 | | 5.06 | | | 1.40 | | 4.14 | | 9.59 | | 4.38 | | 4.68 | | 2.62 | |  |  |  |
| Pear Drops | 5.66 | | 5.00 | | 4.74 | | 3.08 | | 4.56 | | | 1.58 | | 4.12 | | 8.89 | | 4.02 | | 4.16 | | 2.28 | |  |  |  |
| Peppermint | 5.56 | | 5.18 | | 4.64 | | 3.16 | | 4.98 | | | 1.08 | | 3.92 | | 10.66 | | 4.51 | | 4.70 | | 2.55 | |  |  |  |
| Pineapple | 4.69 | | 4.35 | | 4.53 | | 3.24 | | 3.96 | | | 1.31 | | 4.10 | | 10.43 | | 3.65 | | 3.69 | | 2.36 | |  |  |  |
| Potato | 4.47 | | 5.41 | | 2.82 | | 4.78 | | 3.84 | | | 1.80 | | 4.45 | | 12.94 | | 3.69 | | 3.59 | | 2.11 | |  |  |  |
| Raspberry | 3.88 | | 3.90 | | 4.12 | | 3.40 | | 2.86 | | | 1.32 | | 4.18 | | 13.28 | | 2.86 | | 2.86 | | 1.80 | |  |  |  |
| Rhubarb | 4.22 | | 4.80 | | 3.92 | | 3.98 | | 3.08 | | | 1.56 | | 4.18 | | 12.60 | | 3.00 | | 2.92 | | 1.72 | |  |  |  |
| Rosemary | 4.53 | | 4.96 | | 3.84 | | 3.82 | | 3.94 | | | 0.98 | | 4.27 | | 11.51 | | 3.31 | | 3.53 | | 2.32 | |  |  |  |
| Rum | 4.30 | | 5.64 | | 2.90 | | 4.94 | | 3.50 | | | 1.54 | | 4.52 | | 14.28 | | 2.98 | | 3.28 | | 1.70 | |  |  |  |
| Sage | 4.96 | | 5.24 | | 4.12 | | 3.58 | | 4.24 | | | 0.92 | | 4.38 | | 10.00 | | 3.46 | | 3.69 | | 2.28 | |  |  |  |
| Shea & Butter | 5.43 | | 4.76 | | 4.90 | | 2.96 | | 4.69 | | | 1.22 | | 3.84 | | 9.45 | | 4.69 | | 4.45 | | 2.16 | |  |  |  |
| Spearmint | 5.90 | | 4.96 | | 5.08 | | 2.60 | | 5.72 | | | 1.48 | | 3.56 | | 7.83 | | 5.57 | | 5.40 | | 2.71 | |  |  |  |
| Strawberry | 5.37 | | 4.24 | | 5.37 | | 2.45 | | 4.69 | | | 1.82 | | 3.76 | | 10.64 | | 4.06 | | 4.41 | | 2.22 | |  |  |  |
| Sweet Sherry | 4.58 | | 5.46 | | 3.16 | | 4.62 | | 3.88 | | | 1.48 | | 4.54 | | 12.63 | | 2.98 | | 3.50 | | 2.03 | |  |  |  |
| Tea Leaf | 4.02 | | 4.59 | | 3.80 | | 3.96 | | 3.10 | | | 1.06 | | 4.24 | | 13.27 | | 2.98 | | 2.98 | | 1.72 | |  |  |  |
| Toffee Apple | 4.10 | | 4.60 | | 4.04 | | 3.78 | | 2.96 | | | 1.12 | | 4.22 | | 13.24 | | 3.33 | | 3.10 | | 1.63 | |  |  |  |
| Vanilla | 3.66 | | 3.60 | | 3.76 | | 3.60 | | 3.00 | | | 1.12 | | 3.96 | | 13.52 | | 2.88 | | 2.65 | | 1.81 | |  |  |  |
| Watermelon | 5.34 | | 4.54 | | 5.52 | | 2.50 | | 4.48 | | | 1.80 | | 3.76 | | 9.50 | | 4.08 | | 4.36 | | 2.24 | |  |  |  |
| Whisky | 3.61 | | 5.02 | | 2.59 | | 4.71 | | 3.08 | | | 1.65 | | 4.54 | | 13.83 | | 2.84 | | 2.86 | | 1.76 | |  |  |  |
| Tomato Plant | 4.88 | | 4.78 | | 4.27 | | 3.37 | | 4.02 | | | 0.84 | | 3.98 | | 11.45 | | 4.04 | | 3.65 | | 2.01 | |  |  |  |
| Menthol | 3.30 | | 3.30 | | 3.68 | | 3.74 | | 2.78 | | | 1.00 | | 3.78 | | 14.54 | | 2.72 | | 2.70 | | 1.34 | |  |  |  |
| Methane | 3.57 | | 5.71 | | 1.71 | | 5.65 | | 3.22 | | | 2.29 | | 4.49 | | 13.80 | | 2.55 | | 3.13 | | 1.72 | |  |  |  |
| Mountain Heather | 4.80 | | 4.12 | | 4.47 | | 3.00 | | 4.22 | | | 1.04 | | 3.71 | | 10.64 | | 3.73 | | 3.76 | | 2.45 | |  |  |  |
| Mouse | 3.36 | | 5.06 | | 2.70 | | 4.70 | | 2.78 | | | 1.50 | | 4.76 | | 14.50 | | 2.62 | | 2.38 | | 1.53 | |  |  |  |
| Myrrh | 3.71 | | 4.31 | | 3.86 | | 3.88 | | 3.22 | | | 0.80 | | 4.12 | | 12.85 | | 3.08 | | 2.92 | | 1.63 | |  |  |  |
| Nag Champa | 3.10 | | 3.41 | | 3.73 | | 3.78 | | 2.35 | | | 0.77 | | 4.08 | | 16.08 | | 2.57 | | 2.29 | | 1.28 | |  |  |  |
| New Car | 4.18 | | 3.43 | | 4.61 | | 3.24 | | 3.61 | | | 0.86 | | 3.65 | | 12.00 | | 3.53 | | 3.20 | | 2.01 | |  |  |  |
| Nutmeg | 3.14 | | 4.32 | | 3.24 | | 4.34 | | 2.60 | | | 1.32 | | 4.60 | | 15.22 | | 2.64 | | 2.50 | | 1.25 | |  |  |  |
| Oak | 3.37 | | 4.51 | | 2.90 | | 4.47 | | 2.78 | | | 1.43 | | 4.33 | | 13.61 | | 2.90 | | 2.65 | | 1.76 | |  |  |  |
| Old Drifter (Ship) | 3.39 | | 5.27 | | 2.31 | | 5.10 | | 3.06 | | | 1.82 | | 4.49 | | 14.38 | | 2.67 | | 2.90 | | 1.99 | |  |  |  |
| Old Inn | 3.18 | | 4.12 | | 2.86 | | 4.32 | | 2.50 | | | 1.50 | | 4.18 | | 14.41 | | 2.34 | | 2.42 | | 1.46 | |  |  |  |
| Old Smithy | 4.60 | | 4.72 | | 3.98 | | 3.65 | | 3.94 | | | 1.06 | | 4.24 | | 11.02 | | 3.54 | | 3.28 | | 1.99 | |  |  |  |
| Out At Sea | 4.65 | | 4.67 | | 3.67 | | 3.90 | | 3.33 | | | 1.08 | | 4.10 | | 11.07 | | 3.50 | | 3.29 | | 1.99 | |  |  |  |
| Ozone | 3.92 | | 4.38 | | 3.68 | | 3.82 | | 3.46 | | | 1.28 | | 4.35 | | 11.35 | | 3.34 | | 3.12 | | 1.75 | |  |  |  |
| Patchouli | 3.55 | | 5.06 | | 3.02 | | 4.43 | | 2.67 | | | 1.31 | | 4.78 | | 13.89 | | 2.85 | | 2.47 | | 1.62 | |  |  |  |
| Peat | 3.31 | | 3.27 | | 3.94 | | 3.63 | | 2.78 | | | 0.76 | | 3.76 | | 14.76 | | 2.73 | | 2.38 | | 1.63 | |  |  |  |
| Pencils | 3.02 | | 3.86 | | 3.33 | | 3.94 | | 2.73 | | | 1.04 | | 3.98 | | 14.78 | | 2.61 | | 2.57 | | 1.45 | |  |  |  |
| Pine/Heather/Peat | 4.42 | | 4.64 | | 3.92 | | 3.84 | | 3.50 | | | 1.08 | | 4.56 | | 12.36 | | 3.30 | | 3.16 | | 1.83 | |  |  |  |
| Polish/Wax | 3.94 | | 4.24 | | 4.20 | | 3.51 | | 3.35 | | | 1.18 | | 4.18 | | 12.55 | | 3.16 | | 3.20 | | 1.89 | |  |  |  |
| Practical Man | 4.86 | | 4.48 | | 4.74 | | 3.16 | | 4.10 | | | 1.26 | | 4.28 | | 12.56 | | 3.96 | | 3.72 | | 1.98 | |  |  |  |
| Racing Car | 3.38 | | 6.32 | | 1.88 | | 5.42 | | 2.90 | | | 2.40 | | 4.62 | | 14.18 | | 2.53 | | 2.66 | | 1.45 | |  |  |  |
| Rockpools | 3.61 | | 3.35 | | 4.04 | | 3.65 | | 2.78 | | | 1.14 | | 3.55 | | 13.86 | | 2.69 | | 2.84 | | 1.47 | |  |  |  |
| Roselle | 4.06 | | 4.00 | | 4.20 | | 3.24 | | 3.24 | | | 1.20 | | 4.06 | | 12.23 | | 3.12 | | 3.00 | | 1.88 | |  |  |  |
| Rum Barrel | 3.10 | | 5.18 | | 2.68 | | 4.66 | | 2.54 | | | 1.56 | | 4.66 | | 15.65 | | 2.46 | | 2.50 | | 1.26 | |  |  |  |
| Sandalwood | 2.73 | | 2.96 | | 3.73 | | 3.69 | | 2.27 | | | 0.69 | | 3.83 | | 14.90 | | 2.50 | | 2.13 | | 1.17 | |  |  |  |
| Sea Breeze | 4.46 | | 4.38 | | 4.14 | | 3.62 | | 3.92 | | | 1.12 | | 4.04 | | 11.29 | | 3.94 | | 3.76 | | 2.18 | |  |  |  |
| Sea Mineral | 5.20 | | 4.39 | | 4.53 | | 3.33 | | 4.41 | | | 1.18 | | 4.06 | | 10.90 | | 4.06 | | 3.79 | | 2.23 | |  |  |  |
| Sea Shore | 2.96 | | 5.20 | | 2.20 | | 5.10 | | 2.29 | | | 1.84 | | 4.81 | | 15.86 | | 2.27 | | 2.08 | | 1.53 | |  |  |  |
| Smugglers | 4.56 | | 5.52 | | 2.12 | | 5.50 | | 3.78 | | | 2.36 | | 3.92 | | 12.89 | | 3.34 | | 3.70 | | 2.17 | |  |  |  |
| Sports Changing Room | 4.16 | | 5.12 | | 3.43 | | 4.06 | | 3.49 | | | 1.06 | | 4.69 | | 12.12 | | 3.10 | | 3.00 | | 1.84 | |  |  |  |
| Sports Rub | 5.60 | | 5.52 | | 4.21 | | 3.29 | | 5.19 | | | 1.08 | | 4.19 | | 8.52 | | 4.45 | | 4.38 | | 2.69 | |  |  |  |
| Stars Dressing Room | 4.38 | | 4.06 | | 4.49 | | 3.30 | | 3.89 | | | 1.17 | | 4.04 | | 11.33 | | 3.53 | | 3.43 | | 1.95 | |  |  |  |
| Sun tan lotion | 5.16 | | 4.22 | | 5.00 | | 2.80 | | 4.64 | | | 1.52 | | 4.10 | | 8.92 | | 4.42 | | 3.90 | | 2.22 | |  |  |  |
| Tarmac | 3.10 | | 6.19 | | 1.96 | | 5.67 | | 2.64 | | | 2.29 | | 5.21 | | 14.86 | | 2.38 | | 2.85 | | 1.86 | |  |  |  |
| Tea Tree Oil | 5.20 | | 5.14 | | 3.67 | | 3.80 | | 4.04 | | | 1.18 | | 4.47 | | 10.72 | | 3.65 | | 3.86 | | 2.20 | |  |  |  |
| Tobacco Leaf | 3.75 | | 4.73 | | 3.10 | | 4.44 | | 3.00 | | | 1.35 | | 4.33 | | 13.74 | | 3.13 | | 2.77 | | 1.88 | |  |  |  |
| Toothpaste | 3.80 | | 4.94 | | 3.37 | | 4.22 | | 3.20 | | | 1.45 | | 4.27 | | 12.76 | | 3.27 | | 3.12 | | 1.84 | |  |  |  |
| Train Smoke | 3.96 | | 6.06 | | 2.18 | | 5.30 | | 3.37 | | | 2.10 | | 4.68 | | 12.31 | | 2.92 | | 2.94 | | 2.39 | |  |  |  |
| Trophy Room | 2.94 | | 4.46 | | 2.98 | | 4.60 | | 2.17 | | | 1.35 | | 4.58 | | 15.18 | | 2.25 | | 2.15 | | 1.25 | |  |  |  |
| Turpentine | 4.33 | | 4.59 | | 3.65 | | 3.69 | | 3.45 | | | 0.96 | | 4.24 | | 12.41 | | 3.39 | | 3.13 | | 1.78 | |  |  |  |
| Tyres | 4.00 | | 5.80 | | 2.18 | | 5.10 | | 3.71 | | | 1.86 | | 5.06 | | 12.61 | | 2.86 | | 3.08 | | 1.99 | |  |  |  |
| Victorian Street | 2.98 | | 5.59 | | 1.86 | | 5.49 | | 2.39 | | | 2.31 | | 4.57 | | 14.92 | | 2.31 | | 2.27 | | 1.62 | |  |  |  |
| Washday | 4.78 | | 4.86 | | 3.96 | | 3.61 | | 4.02 | | | 1.08 | | 3.94 | | 10.67 | | 3.86 | | 3.57 | | 1.95 | |  |  |  |
| Washing Up Liquid | 5.65 | | 4.71 | | 5.33 | | 2.65 | | 4.80 | | | 1.75 | | 3.78 | | 9.64 | | 4.46 | | 4.35 | | 2.45 | |  |  |  |
| Wood Chip | 2.94 | | 3.96 | | 3.80 | | 3.98 | | 2.42 | | | 1.16 | | 4.12 | | 14.60 | | 2.66 | | 2.28 | | 1.18 | |  |  |  |
| Woodsmoke | 4.28 | | 5.00 | | 2.94 | | 4.88 | | 3.62 | | | 1.50 | | 4.28 | | 14.54 | | 3.06 | | 3.22 | | 2.11 | |  |  |  |
| Ylang, Jasmine & Myrrh | 4.80 | | 4.56 | | 4.60 | | 3.18 | | 4.02 | | | 0.96 | | 4.22 | | 10.33 | | 4.30 | | 3.74 | | 2.24 | |  |  |  |
| Aftershave | 4.96 | | 4.24 | | 4.74 | | 3.02 | | 4.34 | | | 1.06 | | 4.04 | | 12.19 | | 4.22 | | 3.78 | | 2.31 | |  |  |  |
| Soap Suds | 4.90 | | 4.14 | | 4.37 | | 3.22 | | 4.63 | | | 1.10 | | 3.53 | | 9.60 | | 4.29 | | 4.10 | | 2.54 | |  |  |  |
| Rubber | 4.12 | | 5.80 | | 2.22 | | 5.39 | | 3.90 | | | 1.86 | | 4.98 | | 12.06 | | 3.17 | | 3.16 | | 2.15 | |  |  |  |
